# Landscape-Level Drivers of Fungal Communities in Grapevine, Fruit Trees, and Semi-Natural Shrublands in a Habitat Matrix

**DOI:** 10.3390/plants14203178

**Published:** 2025-10-16

**Authors:** Luca Annamária Lepres, Anna Molnár, Adrienn Geiger, Kálmán Zoltán Váczy, József Geml

**Affiliations:** 1 HUN-REN-EKKE Lendület Environmental Microbiome Research Group, Eszterházy Károly Catholic University, H-3300 Eger, Hungary; molnar.anna@uni-eszterhazy.hu (A.M.); geiger.adrienn@uni-eszterhazy.hu (A.G.); vaczy.kalman@uni-eszterhazy.hu (K.Z.V.); geml.jozsef@uni-eszterhazy.hu (J.G.); 2 Food and Wine Research Centre, Eszterházy Károly Catholic University, H-3300 Eger, Hungary; 3 Doctoral School of Environmental Sciences, Hungarian University of Agriculture and Life Sciences, H-2100 Gödöllő, Hungary

**Keywords:** *Rosaceae* family, fungal communities, host plant identity, grapevine trunk diseases

## Abstract

The grapevine microbiome is shaped by a complex interplay of biotic and abiotic factors, affecting microbial community structure and plant health. This study investigates the diversity, composition, and dynamics of fungal communities associated with grapevine (*Vitis vinifera*) and neighboring cultivated plants, as well as plants from semi-natural vegetation, including pear (*Pyrus communis*), apricot (*Prunus armeniaca*), dogrose (*Rosa canina*), and blackthorn (*Prunus spinosa*), in a landscape-level habitat matrix. Using metabarcoding techniques, fungal communities from leaves and woody tissues of grapevine and neighboring plants were analyzed over a growing season. Fungal richness and abundance differed significantly among host plants, with woody tissues exhibiting higher diversity. Host plant identity was the primary factor shaping wood-associated fungal communities (15.7% of explained variance), whereas sampling time dominated in leaves (16.3%), with sampling site having a weaker effect in both cases. Pathogenic fungi associated with grapevine trunk diseases, such as *Diaporthe*, *Eutypa*, and *Phaeomoniella*, were identified across grapevine and neighboring plants, suggesting that multiple hosts may act as reservoirs for fungal inoculum. These findings highlight the complex interactions between fungal communities, host plants, and environmental factors, underscoring the need for landscape-level approaches to plant protection that account for both cultivated and surrounding ecosystems.

## 1. Introduction

The vineyard microbiome is continuously shaped by spatial and temporal variations and by the complex interplays of biotic and abiotic factors [1,2,3,4,5]. Climate and weather patterns play a crucial role in driving the spatial heterogeneity and the year-round exchange of microbiota associated with grapevine and their environment. Various potential reservoirs, such as soil, vine bark, and neighboring plants, provide overwintering habitats for grapevine-associated fungi and bacteria [6,7,8]. Microbial community dynamics and composition are further influenced by human activities, insect interactions, and weather events, particularly during the growing seasons. Throughout this process, the plant genotype influences the selection of specific microbiota from the surrounding microbial pool [9,10].

Several metabarcoding studies have analyzed the DNA of microbial communities associated with grapevines and fruit crops. Early research in vineyards suggested that soil serves as a major reservoir for both belowground and aboveground microbiomes [2] and may also harbor pathogens associated with diseases such as esca [11]. However, contrasting results from a Hungarian study found that grapevine trunk disease (GTD)-associated pathogens are rarely detected in soil, implying that infection is unlikely to originate from this compartment [12]. Microbial communities have also been extensively studied in various aboveground grapevine tissues, including trunks, leaves, and berries. These studies found no significant differences in fungal diversity across different vineyard sites, suggesting that geographical location has limited influence on the aboveground mycobiome [4,13,14,15]. Instead, the plant compartment was consistently identified as the primary factor shaping microbial community structure [2,5,12,16]. This is likely due to environmental selection pressures, where each plant part acts as a unique ecological niche favoring specific microbial taxa [10]. Nonetheless, microbial composition can also be influenced by temporal dynamics. Significant shifts in community structure have been observed between seasons and years [15,17]. While a core fungal community is often shared across vineyards, these temporal changes and compartment-specific selections complicate the role of terroir in shaping the grapevine microbiome. However, the “terroir” effect is likely not driven by aboveground microbial communities, as samples from different vineyards showed similar fungal assemblages [15]. Compared to the well-studied soil and root microbiomes, less is known about the origins of microbial communities in aboveground tissues. Leaf-associated microbiota, in particular, may be shaped by neighboring vegetation, as microbial propagules can be transferred via wind or insect carriers [18,19]. Some winegrowers have noted that surrounding plants influence the sensory properties of wine, and compounds from nearby vegetation have indeed been detected in wine samples [19]. Yet, the interactions between the microbiomes of cultivated plants and adjacent native or cultivated flora remain poorly understood [20,21]. Expanding microbiome studies to consider landscape-level microbial exchange may therefore provide new insights into the ecological factors that shape plant health and disease susceptibility.

Despite the increasing use of DNA methods in plant pathology, landscape-level approaches studying microbial communities in a variety of hosts growing in the vicinity of each other are scarce. Yet, it is well established that plant pathogens can spread from a reservoir species to neighboring vegetation [22,23,24]. Grapevine and fruit trees belonging to the *Rosaceae* family, such as pome and stone fruit trees, share many fungal pathogens, e.g., the ones that cause trunk diseases on woody hosts. For example, *Phaeoacremonium* species, which are known to cause esca-type grapevine trunk disease, also colonize apple and apricot trees [25,26,27]. Another GTD pathogenic fungus, *Eutypa lata*, has also been reported from a number of other cultivated and native woody species, including *Prunus avium*, *Prunus cerasus*, *Prunus dulcis*, *Malus domestica*, *Pyrus communis,* and *Juglans regia* [28,29], although the fungus is most commonly reported to cause disease in apricot and cherry orchards as well as in vineyards [30,31].

To address the above knowledge gap, this study investigates the landscape-scale connections among fungal communities associated with grapevines and those in woody plants dominating the areas surrounding vineyards, such as orchards and regenerating semi-natural shrublands. More specifically, the mycobiomes of leaves and woody tissues of grapevine, selected fruit tree species, and naturally occurring native rosid shrubs growing in adjacent areas were compared. Thus, this study applied a regional approach to examine not only the economically significant plant but also the semi-natural vegetation growing in its environment. Additionally, the composition, diversity, and dynamics of the microbiomes of cultivated and native plants were compared over a growing season in two heterogeneous landscapes composed of the above-mentioned matrix elements.

Vineyards and fruit orchards are managed agricultural ecosystems characterized by low plant species and microbial diversity, where weeds, pests, and pathogens are regularly controlled by various cultural practices and chemicals to preserve crops and to reduce leaf and fruit infections. In contrast, semi-natural shrublands are unmanaged habitats representing early stages of natural secondary succession, typically supporting higher biodiversity and more complex microbial communities. Therefore, it is expected that fungal communities associated with native rosid shrubs in semi-natural habitats would be richer than neighboring vineyards and fruit orchards (Hypothesis 1). Additionally, it is hypothesized that the distance from adjacent shrubland affects the composition of fungal communities in vineyards, as proximity to these semi-natural habitats may facilitate dispersal or establishment of fungal taxa (Hypothesis 2). Moreover, it is assumed that the compositional dynamics of fungi would be driven mainly by site characteristics, such as mesoclimate, vs. biotic factors, e.g., host plant identity (Hypothesis 3). In addition, because endophytic fungal communities tend to differ greatly among plant parts [5,12], compositional differences among leaves and woody tissues are expected in all hosts, and it is hypothesized that seasonality affects the leaf and wood microbiomes differently (Hypothesis 4). Finally, based on known shared trunk disease pathogens among grapevine and fruit trees as mentioned above, it is expected that this environmental DNA study would reveal a broad range of shared pathogens (Hypothesis 5).

## 2. Results

A total of 18,287 fungal amplicon sequence variants (ASVs) were identified at the genus level in the leaf samples and 31,058 in the wood samples. Plant pathogenic fungi were represented by 5385 ASVs in leaves and 5679 in wood, including 555 ASVs of GTD-associated plant pathogens in wood samples. 1739 ASVs were assigned to saprophytic fungi in leaves and 4702 ASVs in wood. Litter saprotrophs were represented by 2805 ASVs in leaves, and wood saprotrophs were represented by 1751 ASVs in wood. The genera with the highest number of ASVs in leaves were *Phyllosticta* (571 ASVs), *Phaeosphaeria* (520 ASVs), *Alternaria* (484 ASVs), *Pseudopezicula* (395 ASVs), and *Ascochyta* (328 ASVs). *Alternaria* (1033 ASVs), *Seimatosporium* (752 ASVs), *Cladosporium* (583 ASVs), *Phaeomoniella* (304 ASVs), and *Dothiora* (304 ASVs) were the most dominant genera in wood.

### 2.1. Fungal Richness and Abundance in Wood and Leaf Microhabitats

**Figure 1:**
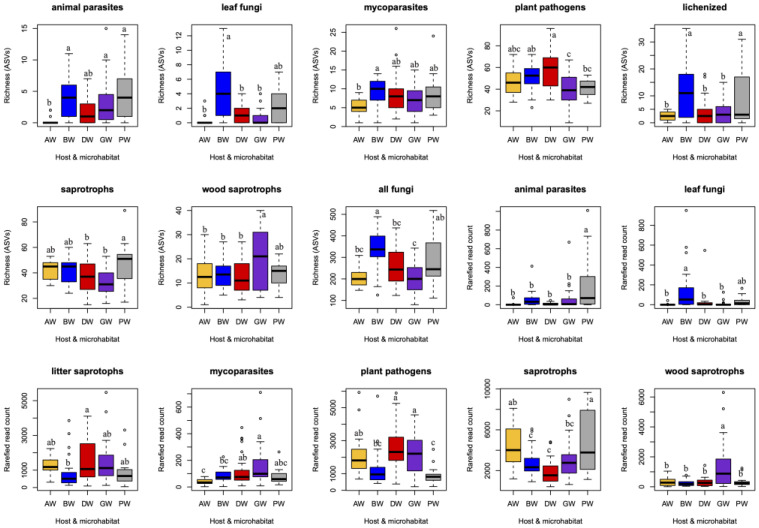
Boxplots showing the ASV richness and rarefied read abundance of functional groups of fungi among wood samples of examined plants, based on the rarefied dataset. Letters indicate significant differences in one-way ANOVA post hoc Tukey HSD test (*p* < 0.05). Abbreviations: W—wood sample, A—apricot, B—blackthorn, D—dogrose, G—grapevine, P—pear.

ASV richness and abundance values of fungal communities associated with different host plants in different microhabitats (wood and leaves) showed notable differences. In wood samples, ASV richness showed significant differences among host plants in all functional groups, except for litter saprotrophs (*p =* 0.0541). In contrast, leaf samples differed significantly only for one functional group, namely leaf fungi (*p =* 0.0029). There were no significant differences in leaf samples abundance among host plants in any functional group, while for wood samples, there were significant differences among host plants in all functional groups except lichenized (*p =* 0.0659). The results of the woody parts by function group are presented in Figure 1.

The species richness of animal parasites was lowest in apricot, and the other host plants had a similar number of sequences, while their abundance was highest in pear and showed uniformly low abundance in other plants. The richness of leaf fungi was highest in blackthorn and pear; this was consistent with the abundance values, where blackthorn and pear samples were also the most abundant. For mycoparasites, samples contained similar amounts of sequences, while apricot and blackthorn showed significant differences (BW-AW: *p =* 0.0082). In contrast, abundance was the highest in grapevine and the lowest in apricot (GW-AW: *p =* 0.0009). However, there was also a significant difference between apricot and dogrose (AW-DW: *p =* 0.0279) and grapevine and blackthorn (GW-BW: *p =* 0.0307). Plant pathogen richness was the highest in dogrose and the lowest in pear and grapevine (DW-GW: *p =* 0.0001; DW-PW: *p =* 0.0024; BW-GW: *p =* 0.0074). While abundance was high in both grapevine and dogrose, the lowest was observed in pear (DW-PW: *p =* 0.0001; GW-PW: *p =* 0.0118). Lichenized sequence richness was highest in blackthorn and pear, with significantly fewer sequences observed in apricot, dogrose, and grapevine. In this case, no significant differences were observed in abundance between plants in the lichenized group. Saprotroph richness revealed significant differences between pear and grapevine (PW-GW: *p =* 0.0036) and between pear and dogrose (PW-DW: *p =* 0.0485), and richness and abundance were the highest in pear. Wood saprotrophs showed the highest values in the grapevine in both cases. Litter saprotrophs showed significant differences only in abundance. In this case, significant differences were detected in the abundance of dogrose and blackthorn (DW-BW: *p =* 0.0333).

### 2.2. Effect of Shrub Proximity on Grapevine Fungal Communities

**Figure 2:**
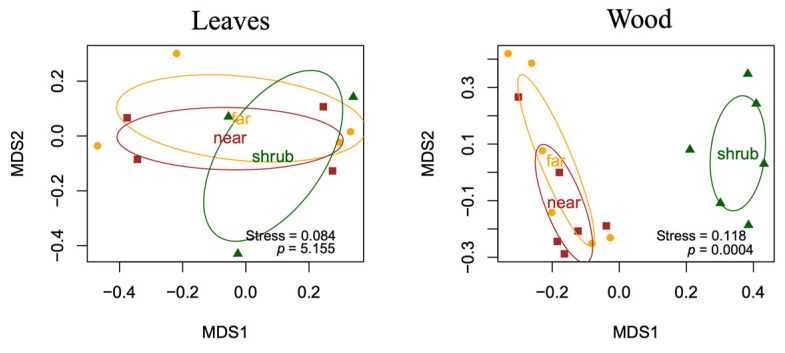
Non-metric multidimensional scaling (NMDS) ordination plots showing the differences in composition of the fungal communities in leaves and wood tissues of grapevine (near and far) and shrubs (blackthorn and dogrose). The ordination was based on a Bray–Curtis distance matrix generated from the abundance table.

To investigate the effect of distance from the shrubland, the composition of fungal communities in grapevine leaves and woody tissues was analyzed using non-metric multidimensional scaling (NMDS). The effect of shrubland proximity (near: 5 m, far: 80 m) was tested by comparing the fungal communities of grapevines located close to and farther from the shrubland. When comparing near and far grapevines separately, no statistically significant differences were found in either the leaves or the woody tissues. According to the PERMANOVA test, distance from the shrubland explained 9.2% of the variation in leaf-associated fungal communities (*p* = 0.858) and 8.1% of the variation in wood-associated communities (*p* = 0.5085). These results indicate that, at the spatial scale investigated, proximity to shrubland was not a significant factor shaping the composition of fungal communities. In contrast, the NMDS analysis revealed that fungal communities in woody tissues differed significantly between the shrub and the grapevines (PERMANOVA: R^2^ = 24.2%, *p* = 0.0004) (Figure 2).

However, the communities of grapevines located closer to or farther from the shrub were similar to each other, suggesting that distance from the shrub did not substantially influence community structure. The observed difference is therefore primarily attributable to the host plant species: the composition of fungal communities in woody tissues was determined more by the plant species itself than by the grapevines’ position relative to the shrub. The fungal community composition of leaves did not differ significantly between the shrub and the grapevines (PERMANOVA: R^2^ = 18.6%, *p* = 0.5155) (Figure 2). The lack of significant separation indicates that, in contrast to woody tissues, neither the host plant species nor the distance from the shrub strongly influenced the structure of foliar fungal communities. Thus, fungal communities in leaves appear to be more homogeneous across host plant types and positions relative to the shrub.

### 2.3. Effects of Host, Site, and Season on Fungal Community Composition in Leaves and Wood

**Figure 3:**
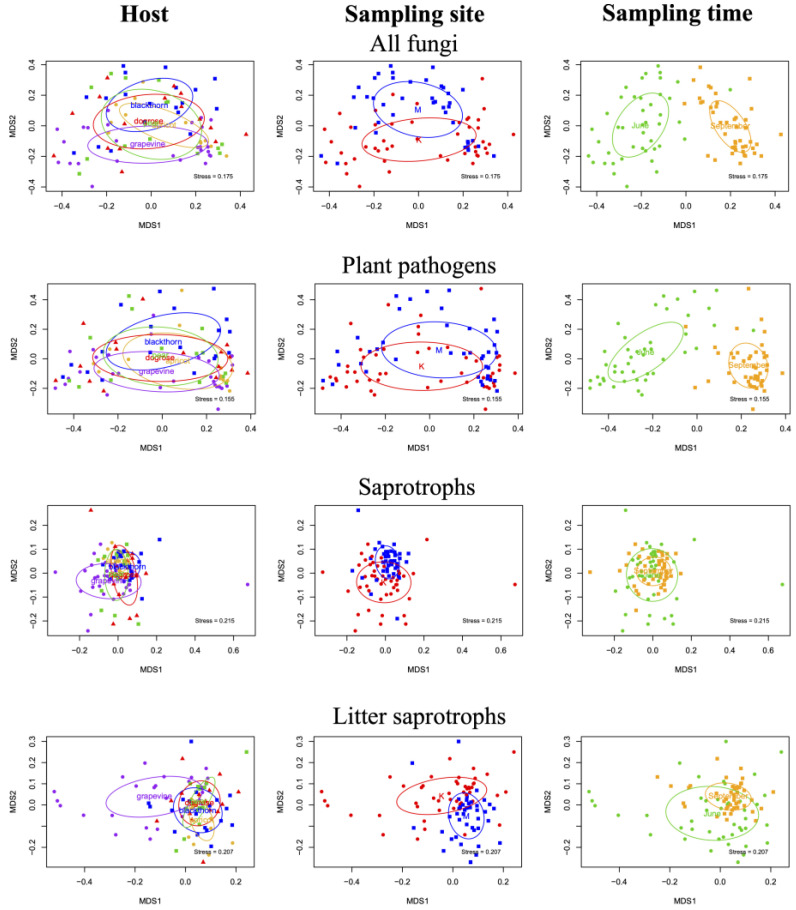
Non-metric multidimensional scaling (NMDS) ordination plots showing the differences in composition of the dominant fungal communities in leaves affected by different influencing factors: host plant, sampling site (“K” means Kőlyuktető vineyard, “M” means Mihálynagytető vineyard), and sampling time. The ordination was based on a Bray–Curtis distance matrix generated from the abundance table.

**Figure 4:**
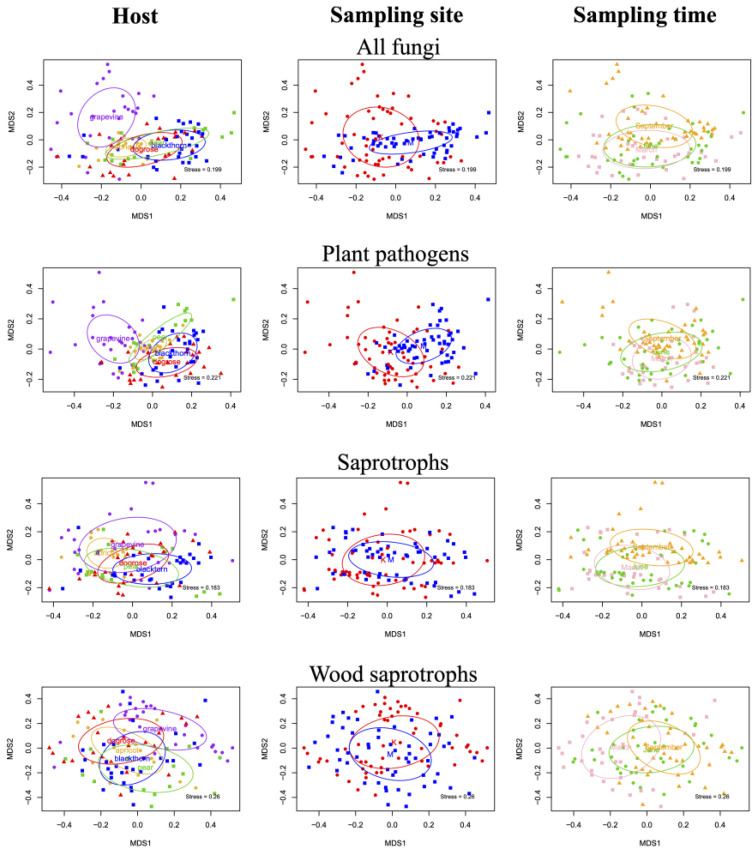
Non-metric multidimensional scaling (NMDS) ordination plots showing the differences in composition of the dominant fungal communities in woody parts affected by different influencing factors: host plant, sampling site (“K” means Kőlyuktető vineyard, “M” means Mihánynagytető vineyard), and sampling time. The ordination was based on a Bray–Curtis distance matrix generated from the abundance table.

The fungal community composition of dominant functional groups, such as plant pathogens, saprotrophs, litter saprotrophs, and wood saprotrophs, was visualized in a two-dimensional NMDS ordination (Figure 3 and Figure 4). The results of PERMANOVA show that all three factors (host plant, sampling site, sampling time) significantly influenced the composition of fungal communities, both for all fungi and for the dominant functional groups considered separately. The results of all three independent variables were significant for all functional groups tested; the compositional variance results and *p*-values are summarized in Table 1.

In the leaves, sampling date, which occurred in two different seasons (summer and autumn), had the largest effect on fungal composition variation, explaining 16.3% of the variance (Figure 3).

Of the three factors, host plant was the second most influential (R^2^ = 10.3%, *p* = 0.0001), and although less influential, site was still a significant factor (R^2^ = 7%, *p* = 0.0001). This pattern was also evident for plant pathogens. This is because, again, sampling time was by far the most determinant factor, explaining 26% of the variance. This was followed by host plant (R^2^ = 7.6%, *p* = 0.0174) and then sampling site (R^2^ = 4.5%, *p* = 0.0017). In contrast, for saprotrophs, the host plant was the dominant factor, explaining 9.8% of the compositional variance. Sampling time was the least influential factor (R^2^ = 4.7%, *p* = 0.0001), and site was the second most influential (R^2^ = 6.3%, *p* = 0.0001). This trend changed again for the litter saprotrophs, where, after host plant (R^2^ = 12.4%, *p* = 0.0001), sampling time (R^2^ = 8.4%, *p* = 0.0001) was the most influential factor, followed by site (R^2^ = 6.5%, *p* = 0.0001).

In woody parts, the host effect was the most influential on fungal community composition (R^2^ = 15.7%, *p* = 0.0001), followed by the sampling site (R^2^ = 5.1%, *p* = 0.0001) and sampling time (R^2^ = 4.8%, *p* = 0.0001) (Figure 4).

The composition of the phytopathogenic fungal communities from woody parts was also strongly influenced by the identity of the host plants, which explained 13.6% of the compositional variance, but in this case, sampling site and sampling date were almost equally important, explaining 5.4% of the compositional variance for sampling area and 5.5% for sampling date. The composition of saprotrophs was most strongly determined by the host plant in the wood samples (R^2^ = 12.4%, *p* = 0.0001). This was followed by the sampling time (R^2^ = 7.7%, *p* = 0.0001) rather than the area, as was the case for leaves. For wood saprotrophs, host plant (R^2^ = 11.8%, *p* = 0.0001) was also the determinant of fungal community composition, followed by sampling time (R^2^ = 5.6%, *p* = 0.0001) and finally site (R^2^ = 2.7%, *p* = 0.0003).

Based on the results of the PERMANOVA analysis, host identity had the strongest influence on the composition of fungal communities in woody tissues. Although host species also had a significant effect on leaf-associated fungi, seasonality was the primary factor shaping their composition. The sampling site also explained a significant proportion of the variation in fungal community composition in both leaf and wood samples, though its influence was less pronounced.

### 2.4. Shared GTD Pathogens in Grapevine and Neighboring Plants

**Figure 5:**
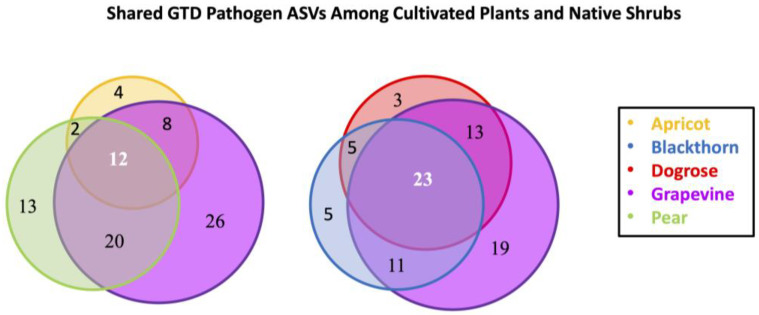
Venn diagram illustrating the number of ASVs—representing GTD pathogens—shared between the cultivated plants, namely grapevine, apricot, and pear, and between grapevine and native shrubs: dogrose and blackthorn.

The Venn diagram shows the overlap of grapevine trunk disease pathogens in wood samples of grapevine and cultivated plants (apricot, pear), as well as grapevine and native shrubs (dogrose, blackthorn) (Figure 5).

A total of 85 ASVs representing GTD pathogens were found in the cultivated plants. Of these, 12 ASVs were common in grapevine, apricot, and pear, representing 14% of the total. When comparing the host plants in pairs, the highest overlap was between pear and grape (23.5% overlap), followed by grape and apricot (9.4% overlap) and finally apricot and pear (2.4% overlap). The following genera were shared among grapevine, pear, and apricot: *Diaporthe*, *Eutypa*, *Phaeomoniella*, *Phaeoacremonium*, and *Neofusicoccum*.

Comparing grapevines with native shrubs, a total of 79 ASVs represented GTD pathogens in the three tested plants, of which 23 ASVs were common (29% overlap). Comparing plants in pairs, the highest overlap was between grapevine and dogrose (16.5% overlap), followed by grapevine and blackthorn (14% overlap), and then blackthorn and dogrose with 6.3% overlap. Overall, the following genera were shared among grapevine, dogrose, and blackthorn: *Diaporthe*, *Phaeomoniella*, *Neofusicoccum*, and *Truncatella*.

## 3. Discussion

This is the first study to explore the relationships of wood and leaf fungal communities among grapevine, fruit orchards, and semi-natural habitats surrounding vineyards, focusing on how host plant identity and abiotic factors such as growing area and season, shape complex fungal communities. The results of this study highlight several key patterns in the fungal communities associated with grapevines and other host plants. Firstly, fungal diversity in intensively managed crop plants (grapevine), extensively managed crop plants (fruit trees), and semi-natural shrubs was found to be comparably high, thereby largely contradicting Hypothesis 1. However, fungal diversity in the woody tissues of blackthorn was significantly higher than in other plants, except for pear. Secondly, no effect of distance from the shrubland on the fungal community composition in grapevines was observed, which contradicts Hypothesis 2. Thirdly, no support was found for Hypothesis 3, which predicted that fungal community composition would be shaped mainly by site characteristics rather than host plant identity. Instead, while the site had a weak but significant effect, host identity was a stronger predictor of differences in fungal communities in leaves and woody tissues alike. Compositional differences between leaves and woody tissues (*p* < 0.0001) were detected, confirming Hypothesis 4. Different environmental factors seemed to drive leaf vs. woody tissue communities: while the composition of wood-inhabiting fungi was primarily shaped by host identity, leaf fungi showed the greatest compositional turnover with seasonality. Finally, several fungal pathogens known to be associated with trunk diseases in grapevines were detected in the woody parts of all studied host plant species, with substantial overlap in ASVs among hosts, supporting our Hypothesis 5.

### 3.1. Patterns of Fungal Richness and Abundance in Foliar and Woody Tissues

The first hypothesis that the fungal community of semi-natural habitats is richer than that of neighboring vineyards and orchards was only partly supported by the results. Significant differences in richness were only found in woody parts. A study on differences in the foliar fungal communities of a vineyard adjacent to forest patches found no significant difference in fungal richness between grapevine leaves and leaves of various tree species in forest patches during summer months [32]. This result is in agreement with the results presented here, because no significant differences were detected in fungal richness between leaves collected in June and early September in the plants studied. Another study also found that between forests and vineyards, the foliar fungal diversity indices were similar, but the community structure was different between habitats. The research explained this by concluding that agricultural practices do not affect fungal diversity but do affect the community structure of fungal communities associated with grapevines [3]. As stated above, both abundance and richness showed more variation in woody parts among host plants. This may be due to the permanence of the woody parts and also because the bark is a potentially important overwintering microhabitat for a wide range of fungi [12,33,34]. A study on the diversity of fungal communities in olive trees found similar results when examining the diversity of fungal communities in flowers, fruits, and leaves, which showed higher levels of diversity in leaves compared to flowers and fruits, regardless of the season. Since leaves are always present on the tree, as olive trees are evergreen, they are able to form a balanced community [35]. For deciduous trees and shrubs, this observation is true for the difference between woody parts and leaves, since in this case, woody parts are present regardless of the season.

### 3.2. Drivers of Fungal Community Composition in Leaves and Woody Tissues: Host vs. Environmental Factors

The results showed that the composition of fungal communities in leaves was mainly determined by season, as opposed to the strong host effect found in wood fungi, with smaller, but significant contributions of site characteristics in community compositional changes. The important role of host species in the wood microbiome was also documented by Krah et al. [36], who found that host species have a greater effect on fungal communities in wood than environmental factors. For leaves, this result is not entirely consistent with other studies that examined the composition and dynamics of fungal communities across multiple habitats and sampling times. They found that host plant species and tissue niches play a greater role in leaf fungal community structure than season. Although seasonality has a significant effect on microbial community composition, there is less variation in community composition between seasons than between host species. Season and its interaction with host species explain less variance than host plant species affiliation [37,38]. Based on these results, the answer to the third hypothesis, which investigated whether site characteristics or host plant affiliation have a greater influence on fungal composition dynamics, is that both have a significant effect, but overall, the host plant has a more dominant effect. The finding that the composition of plant pathogenic fungal communities in leaves is more strongly influenced by seasonal changes compared to woody parts has been observed in several studies. For instance, research highlights that fungal communities in leaves tend to show seasonal successional patterns, with the microbiome being reassembled each growing season as new tissues sprout. This dynamic is particularly evident in annual or deciduous plants where fungi recolonize leaves each spring, leading to changes in community structure over time, driven by environmental conditions and host physiology [39,40]. Additionally, fungal pathogen communities associated with senescing leaves exhibit notable seasonal shifts in composition, influenced by temperature, moisture, and leaf senescence stages, which do not affect woody parts to the same extent due to their structural stability and different ecological roles [39]. These findings underscore that while leaves host dynamic fungal communities that shift with seasons, the microbial populations in woody parts are less influenced by such temporal variations. This distinction is key in understanding host–pathogen interactions and fungal ecology across plant structures. Furthermore, the difference in composition of leaves and woody parts may be caused by strong niche-based processes in the composition of the communities, with many fungi specializing in specific plant parts [4,12,13].

The habitat, as a component of the terroir, plays a significant role in shaping fungal communities associated with grapevines [5,12], and the composition of the fungal communities was significantly different between the two sampling sites (Kőlyuktető vineyard and Mihálynagytető vineyard) in this study as well. Since the two vineyards are located close to each other and experience nearly identical weather conditions, climatic factors are unlikely to explain these differences. Instead, it is hypothesized that variations in soil type, topography, vegetation composition, and cultivation practices contribute to the observed differences. This assumption is supported by previous studies demonstrating that fungal community structure is strongly influenced by microclimate, soil properties, vegetation density and diversity, environmental stresses (e.g., pollution, human intervention), and plant protection measures [3,41,42]

### 3.3. Host Tissue Type Outweighs Shrubland Proximity in Shaping Grapevine Fungal Communities

The hypothesis that the proximity of shrubland significantly influences grapevine fungal communities could not be confirmed within the investigated range of 5–80 m, suggesting that this scale may be too narrow to capture dispersal-driven gradients. In the case of leaves, the analysis considered the entire leaf-associated community, including both endophytic and epiphytic components. Accordingly, leaf communities showed no significant differences between the shrub and the grapevine, whereas woody tissues exhibited a clear separation. This indicates that the host plant species is the main determinant of fungal community structure and that tissue-specific patterns differ: fungal communities in woody parts are more strongly host-specific, while leaf communities remain relatively homogeneous, partly due to the dispersal of epiphytic fungi. This pattern is consistent with the analyses described above, which confirm that the host plant strongly shapes fungal communities in woody tissues, while its influence is less dominant in leaves.

### 3.4. Surrounding Plants as Reservoirs of Grapevine Trunk Disease Pathogens

The results showed that the GTD pathogens were present in all the tested plants, but not in equal proportions, suggesting different susceptibility. The significant overlap of grapevine trunk disease pathogens found in the woody tissues of various host plants suggests that all examined species may serve as hosts for the fungal species responsible for GTDs. Consequently, these plants can also act as potential sources of inoculum for grapevines. A limitation of this study is that the DNA-based detection methods employed (e.g., amplicon-based community profiling) do not allow for the distinction between viable, active fungi and dormant or dead fungal cells. Therefore, the detection of a fungal species in plant tissue does not necessarily confirm its current activity or pathogenicity in that host. This limitation must be considered when interpreting the results. However, several previous studies have confirmed that the fungal genera identified in our work are indeed capable of infecting the plant species under investigation. For instance, several known and potentially novel GTD pathogens—including *Phaeoacremonium*, *Neofusicoccum*, and *Eutypa* species—have been isolated from apple, pear, and various *Prunus* species, with pathogenicity confirmed through inoculation trials [25,26,43,44,45]. Additionally, *Truncatella angustata* was reported as the causal agent of leaf spot on dogrose, indicating that this species can persist on non-woody tissues and act as a reservoir for GTD fungi [46]. While no specific studies are available for blackthorn, the frequent identification of GTD-associated fungi on other *Prunus* species implies that blackthorn may similarly serve as an ecological reservoir.

Overall, the results suggest that GTD pathogens can be associated with the tested plant species, from which they may potentially spread to neighboring vineyards. Therefore, disease control strategies should not be limited to the vineyard level alone. It is important to highlight that most GTD pathogens are ubiquitous in grapevines and often remain asymptomatic. They are believed to behave as opportunistic pathogens, becoming active under host stress, which may be triggered by environmental or management-related factors, or by interactions with other pathogens [5,12]. Due to this latent presence, improving cultivation practices—not only for yield and quality but also to enhance plant resilience—will be essential to mitigate GTD-related damage.

## 4. Conclusions

This study provides novel insights into the complex interactions shaping fungal communities in grapevines, fruit orchards, and adjacent semi-natural habitats. Together, these results advance our understanding of how host identity, environmental factors, and plant structure shape fungal communities and pathogen dynamics in vineyard ecosystems. By demonstrating the microbiological connectivity of grapevines with surrounding vegetation, this study emphasizes the importance of landscape-level approaches for sustainable disease management and vineyard health.

## 5. Materials and Methods

### 5.1. Experimental Design and Sampling

**Figure 6:**
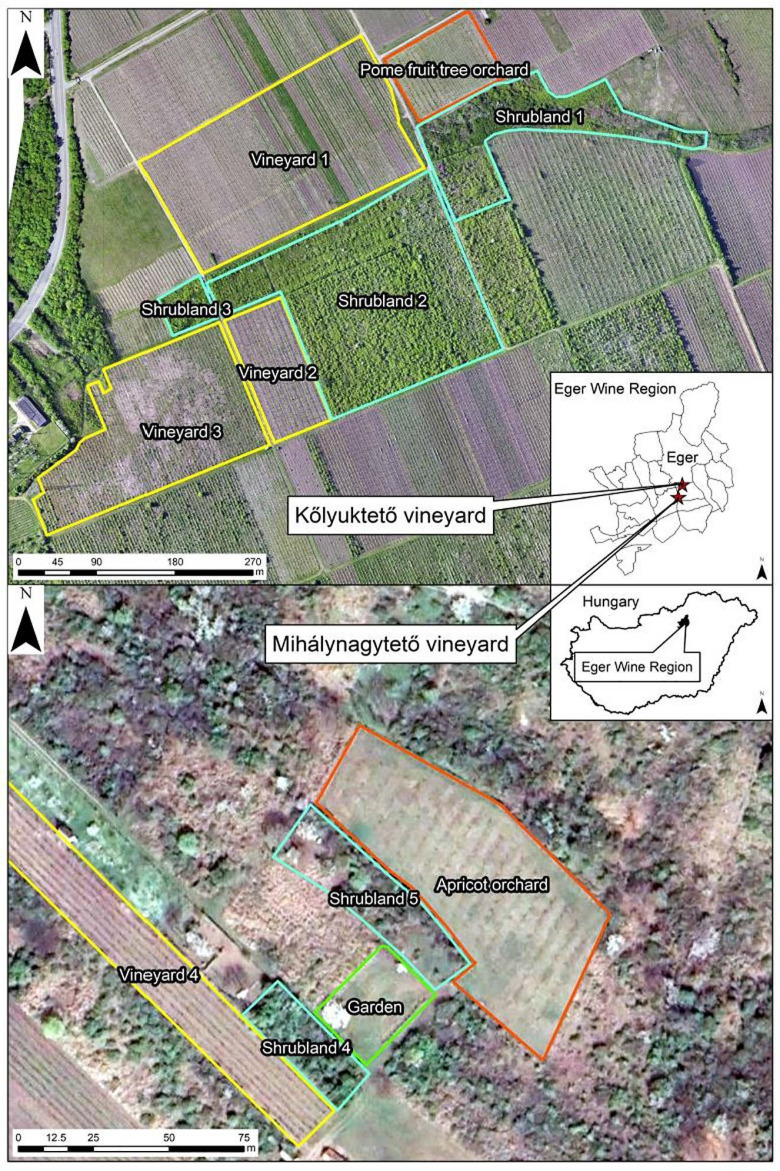
Sampling sites: Kőlyuktető vineyard and Mihánynagytető vineyard.

Randomly chosen grapevine, pear, apricot, dogrose, and blackthorn plants were sampled from two areas in the wine-growing region of Eger, namely the Kőlyuktető vineyard (47.863232N, 20.385180E, above mean sea level: 175 m) and the Mihálynagytető vineyard (47.839640N, 20.371429E, AMSL: 224 m), which are experimental sites of Eszterházy Károly Catholic University (Figure 6).

The research areas are located on the outskirts of the city, primarily in the garden zone on the southern side of Eger, where viticulture developed following medieval deforestation and still exhibits a diverse mosaic-like pattern today. The forested, grassland, or residential patches situated in the immediate vicinity of the vineyards create different ecological conditions, which may influence the structure of microbial communities. There are larger areas dedicated exclusively to vineyards, but there are also many smaller garden plots planted with grapevines or other crops, most of which have a house built on them. Thus, sampling was conducted in well-managed vineyards and orchards embedded in a mosaic landscape, where cultivated areas are interspersed with early successional semi-natural habitats dominated by native shrubs such as dogrose and blackthorn. Fruit trees and grapevines have received conventional plant protection, which, in Hungary, typically includes the regular application of fungicides (e.g., copper- and sulfur-based compounds, systemic fungicides), insecticides, and herbicides according to standard agricultural practice.

Notably, the Kőlyuktető vineyard is situated on brown forest soil with clay illuviation, characterized by distinct soil horizons with clay accumulation in the subsoil, while the Mihálynagytető vineyard is located on leached brown forest soil, which is marked by more pronounced nutrient leaching and generally more acidic conditions. Due to the close proximity of the study areas, no significant differences in weather conditions were expected. Meteorological data, including temperature, precipitation, and humidity for the year 2021, were obtained from the Boreas Plant Protection Station located in the Kőlyuktető vineyard (Supplementary Figures S1–S3).

In both areas, plants that had been marked at the time of the initial sampling were selected. The first sampling was in March of 2021, and we repeated this in June and September. Two different microhabitats were sampled for each plant: leaves and woody tissue, including bark, cambium, and sapwood, collected by cutting a section from a branch. In June and September, samples were collected from leaves and woody parts, but in March, only wood samples were collected due to the lack of leaves. In total, 245 samples were collected from asymptomatic plants in two distinct sampling areas during the 2021 growing season, of which 98 samples were from leaves and 147 from woody parts. A total of 45 grapevine, 33 blackthorn, 30 dogrose, 21 pear, and 18 apricot wood samples were collected on the three sampling dates, and 30 grapevine, 22 blackthorn, 20 dogrose, 14 pear, and 12 apricot leaf samples were collected during the June and September sampling dates. The sample collection strategy is summarised in Supplementary Figure S4.

To investigate the effect of distance from the shrubland on the fungal community in vineyards, grapevine samples were collected in two rows, from plants close to the shrub and two further away from the shrub, from “Vineyard 1” (Figure 6), adjacent to a shrubland on one side. The other adjacent areas were all vineyards or orchards. Sampling in this case was also done in March, June, and September. Nearby grapevines were located 5 m from the shrubland, and those further away were 80 m away.

### 5.2. DNA Extraction, PCR, and Sequencing

Immediately after fieldwork, the samples were transported to the laboratory, where they were stored at −80 °C. They were then lyophilized for 72 h to ensure complete desiccation and subsequently homogenized using a tissue lyser with steel beads. Genomic DNA was extracted from approximately 20 mg of the lyophilized and homogenized plant material from each composite sample using Plant DNA isolation kits (Macherey-Nagel GmbH & Co., Düren, Germany). The concentration and purity of the extracted genomic DNA were assessed using a NanoDrop 2000 spectrophotometer (Thermo Fisher Scientific, Waltham, MA, USA).

The internal transcribed spacer 2 (ITS2) region of rDNA was PCR-amplified from all samples using the primers fITS7 [47] and ITS4 [48], each appended with Illumina overhang adapter sequences (Illumina, San Diego, CA, USA). PCR reactions were performed in 25 μL volumes containing 12.5 μL of KAPA HiFi HotStart ReadyMix (Roche, Basel, Switzerland), 0.5 μM of each primer, 1 μL of template DNA (~10 ng), and nuclease-free water. The PCR amplification conditions were: initial denaturation at 95 °C for 3 min; 25–32 cycles of 95 °C for 30 s, 56 °C for 30 s, and 72 °C for 30 s; followed by a final extension at 72 °C for 5 min. To assign amplicons to their respective samples, a second PCR was performed with the same primers modified to include Illumina Nextera™ DNA CD Indexes (Illumina, San Diego, CA, USA) according to Illumina’s dual-indexing strategy. PCR products were purified using AMPure XP magnetic beads (Beckman Coulter, Brea, CA, USA), quantified with a Qubit™ dsDNA HS Assay Kit (Thermo Fisher Scientific), and equimolarly pooled based on Qubit measurements. The final libraries were quality-checked using an Agilent 2100 Bioanalyzer with a High Sensitivity DNA Kit. Sequencing was performed on an Illumina MiSeq platform with MiSeq Reagent Kit v2 (Illumina, San Diego, CA, USA) (500 cycles), generating 250 bp paired-end reads. All molecular procedures—including PCR amplification, adapter ligation, library preparation, quality control, and sequencing—were carried out by BIOMI Kft. (Gödöllő, Hungary) following Illumina’s standard amplicon sequencing protocols.

### 5.3. Bioinformatic Work

Raw DNA sequences were processed using the dada2 package [49], which, unlike methods clustering sequences into operational taxonomic units (OTUs), robustly removes spurious data while resolving fine-scale sequence variation to generate unique amplicon sequence variants (ASVs). This approach captures both intra- and interspecific genetic variation of fungi in the samples, enabling strain-level analysis of fungal interactions. Based on visual inspection of quality profiles, forward and reverse reads were truncated at 240 and 200 base pairs, respectively, to remove low-quality terminal regions while retaining high-quality sequence data. Reads were then denoised, chimera-filtered, merged, and inferred as ASVs with default settings. Taxonomic assignments were performed using USEARCH v11 [50] against the UNITE reference database (version released 25 July 2023) [51], which includes dynamically delimited fungal species hypotheses. A minimum confidence threshold of 0.8 was applied for taxonomic classification. Fungal ASVs were assigned to putative functional guilds using the curated FungalTraits database [52], with the following modifications: Saprotrophic fungi that did not belong to litter and wood decomposers, such as nectar/sap saprotrophs, sooty molds, soil saprotrophs, and undefined saprotrophs, were treated as generalist saprotrophs primarily utilizing simple carbohydrates, hereafter referred to as “saprotrophs.” Additionally, “epiphytes” and “endophytes,” which are non-pathogenic leaf-associated fungi, were grouped into the category of leaf fungi. The list of all fungal ASVs identified with functional and taxonomic assignments and their distribution among the samples is displayed in Supplementary Table S1 in the Supplementary Files.

### 5.4. Statistical Analyses

Unless otherwise noted, all statistical analyses were conducted in the R software (version 4.1.2; R Foundation for Statistical Computing, Vienna, Austria) environment [53]. Samples with fewer than 1000 fungal sequences were excluded, resulting in 210 samples included in the subsequent analyses. Rarefaction curves of fungal ASVs of these samples are shown in Supplementary Figure S5. The fungal community matrix was normalized (rarefied) by random subsampling to the smallest library size (11,321 reads) per sample using the rrarefy function in the vegan R package (version 2.5–7) [54]. All sequences of fungal ASVs analyzed in this paper have been deposited in GenBank (KIWX01000001-KIWX01004386).

Because fungal communities were significantly (*p* < 0.0001) different in leaf vs. woody tissues, we analyzed leaf and woody tissue communities separately when testing for the effects of hosts, seasons, and site on richness, rarefied abundance, and community composition. To test for significant effects of categorical variables (e.g., host) on ASV richness and abundance of fungal ASVs among the samples, one-way and two-way analysis of variance (ANOVA) models were used. Only variables with a significant effect in one-way ANOVA were included in the two-way models. Significant pairwise differences were identified using Tukey’s honestly significant difference test. Compositional differences among samples were visualized using nonmetric multidimensional scaling (NMDS) with a Bray–Curtis distance measure on the Hellinger-transformed abundance matrix, utilizing the metaMDS function in vegan with 999 permutations. After testing for no differences in dispersion among groups, permutational multivariate ANOVA (PERMANOVA) was performed to estimate the amount of variation explained by host plant, sampling site, and sampling date using the adonis function in vegan with 9999 permutations. These categorical variables were tested for significance independently and in combination, accounting for any correlations among the variables.

The distribution of GTD pathogen ASVs among sampled plants was visualized with Venn diagrams using Biovenn [55]. GTD pathogens colonize the perennial woody tissues of grapevines and infect through pruning wounds or via mechanical injuries [56], which served as the rationale for focusing exclusively on wood samples and omitting leaf tissue from the analysis. Fungal genera known to cause GTDs with previously published reports and confirmations were regarded as GTD-associated genera, based on the literature search [12].

## Figures and Tables

**Table 1:** Proportion of variation (%) in different source types (wood and leaves) of dominant fungal community composition explained by different host plants, sampling sites, and sampling times with permutational multivariate analysis of variance, based on the fungal community matrix.

	**Plant pathogens**				**All fungi**			
	**in leaf samples**		**in wood samples**		**in leaf samples**		**in wood samples**	
	**%**	***p*** **Value**	**%**	***p*** **Value**	**%**	***p*** **Value**	**%**	***p*** **Value**
Host	7.6	0.0174	13.6	0.0001	10.3	0.0001	15.7	0.0001
Site	4.5	0.0017	5.4	0.0001	7	0.0001	5.1	0.0001
Sampling time	26	0.0001	5.5	0.0001	16.3	0.0001	4.8	0.0001
	**Saprotrophs**				**Litter saprotrophs in leaf samples**		**Wood saprotrophs in wood samples**	
	**in leaf samples**		**in wood samples**					
	**%**	***p* value**	**%**	***p* value**	**%**	***p* value**	**%**	***p* value**
Host	9.8	0.0001	12.4	0.0001	12.4	0.0001	11.8	0.0001
Site	6.3	0.0001	2.8	0.0023	6.5	0.0001	2.7	0.0003
Sampling time	4.7	0.0001	7.7	0.0001	8.4	0.0001	5.6	0.0001

## Data Availability

This Targeted Locus Study project has been deposited at DDBJ/ENA/GenBank under the accession KIWX00000000. The plant metagenome targeted locus study (TLS) project has the project accession KIWX00000000. This version of the project (01) has the accession number KIWX01000000 and consists of sequences KIWX01000001-KIWX01004386. A BioProject was also generated (PRJNA1193836).

## Supplementary Materials

The following supporting information can be downloaded at: https://www.mdpi.com/article/10.3390/plants14203178/s1, Supplementary Figure S1: Daily precipitation amounts in 2021; Supplementary Figure S2: Daily relative humidity in 2021; Supplementary Figure S3: Daily minimum, maximum, and average temperatures in 2021; Supplementary Figure S4: Sampling strategy; Supplementary Figure S5: Rarefaction curves; Supplementary Table S1: The list of all fungal ASVs identified with functional and taxonomic assignments and their distribution among the samples.
