# Supplementary figures and images for: Landscape-Level Drivers of Fungal Communities in Grapevine, Fruit Trees, and Semi-Natural Shrublands in a Habitat Matrix
Source: Plants (Basel). 2025 Oct 16;14(20):3178. doi: 10.3390/plants14203178 (PMC12566767; doi:10.3390/plants14203178)

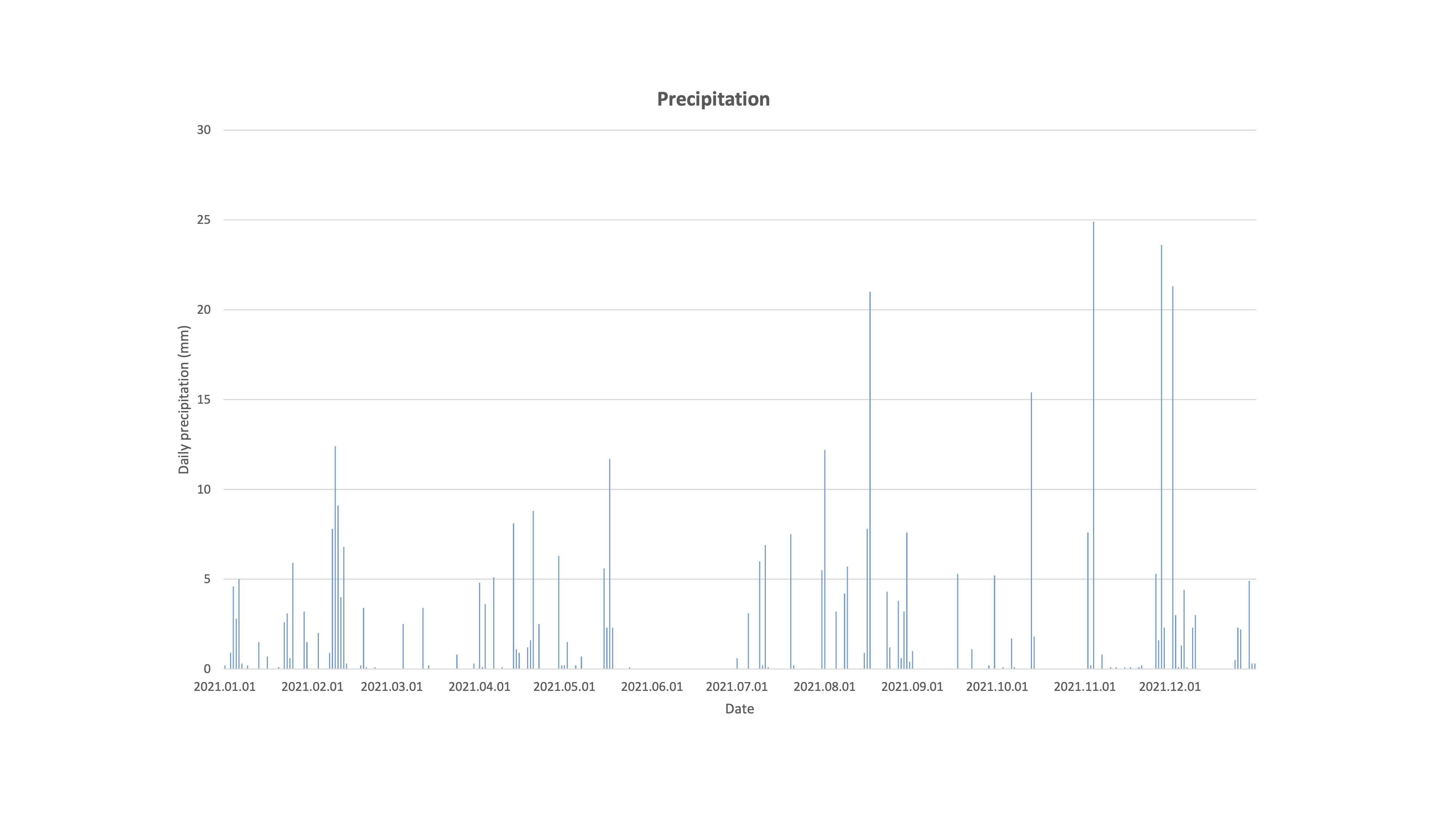

Supplement: Supplementary file 1 [file plants-14-03178-s001.zip › Supplementary_S1.tiff]

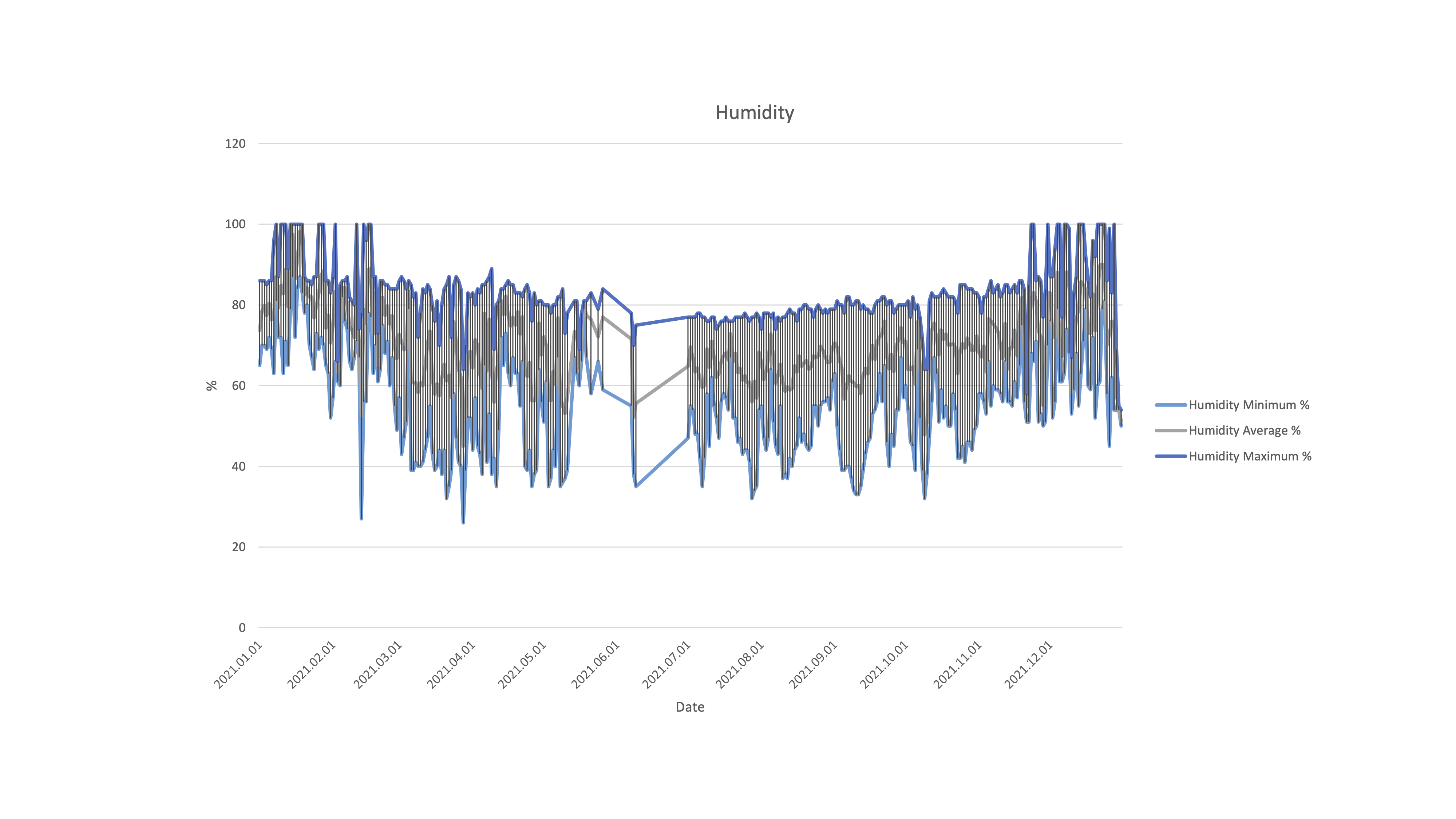

Supplement: Supplementary file 1 [file plants-14-03178-s001.zip › Supplementary_S2.tiff]

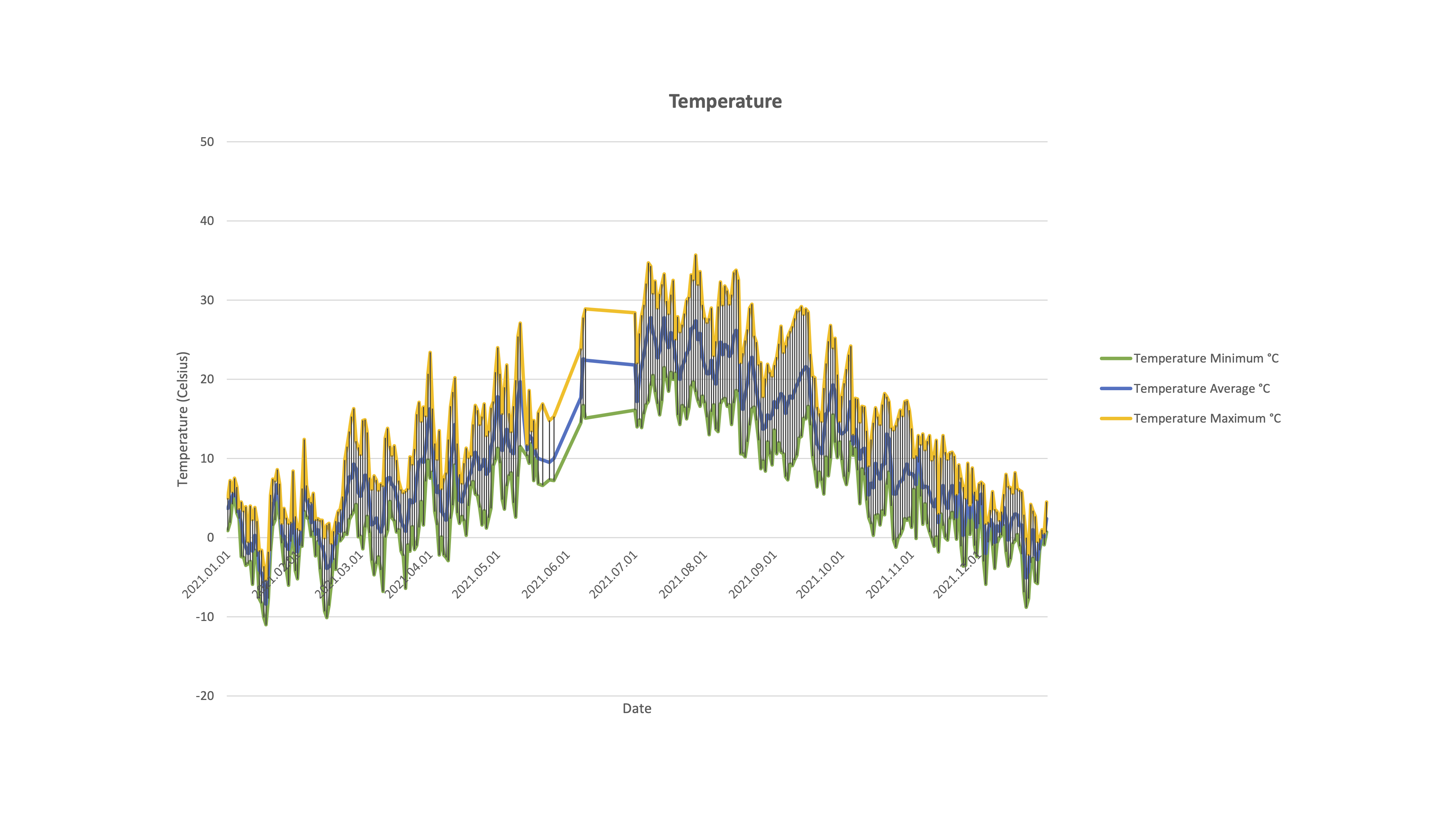

Supplement: Supplementary file 1 [file plants-14-03178-s001.zip › Supplementary_S3.tiff]

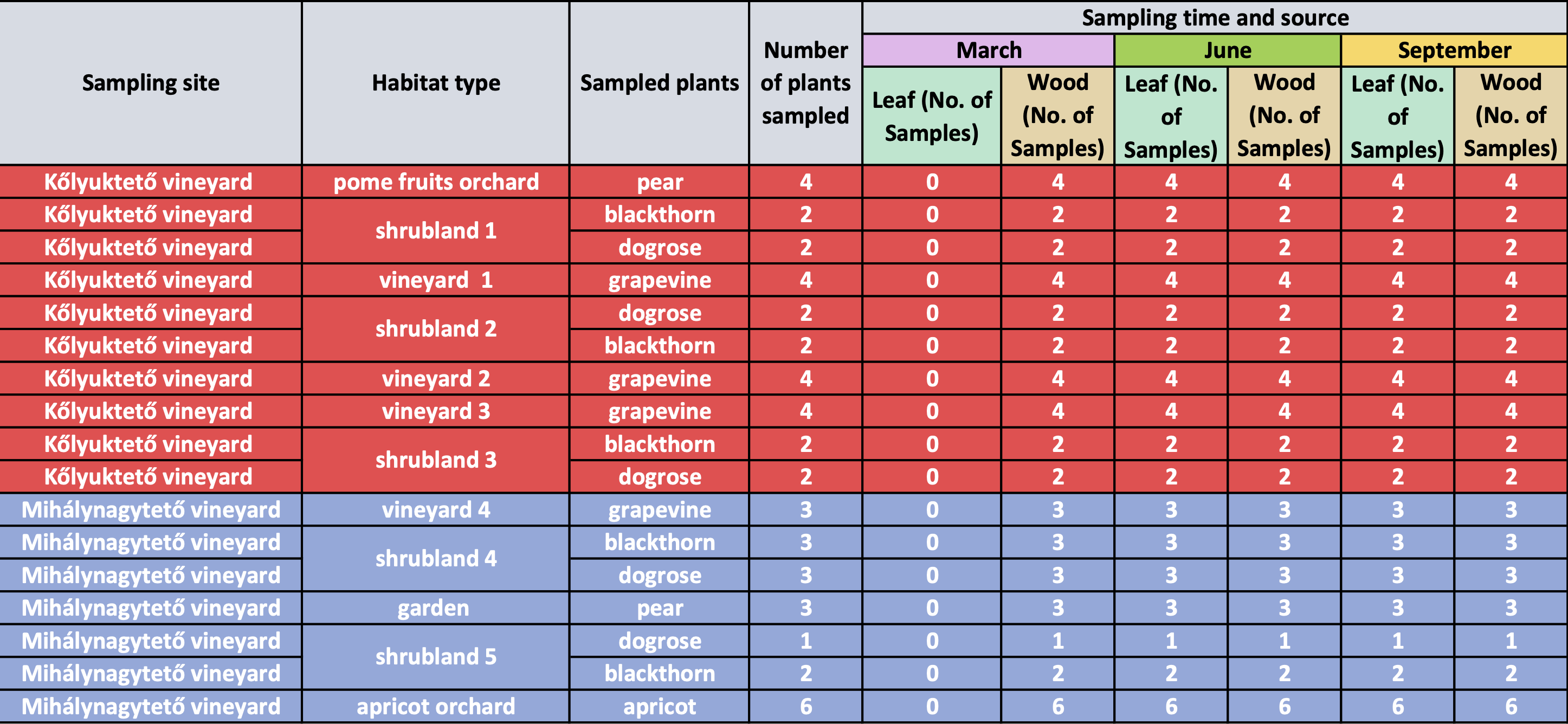

Supplement: Supplementary file 1 [file plants-14-03178-s001.zip › Supplementary_S4.tiff]

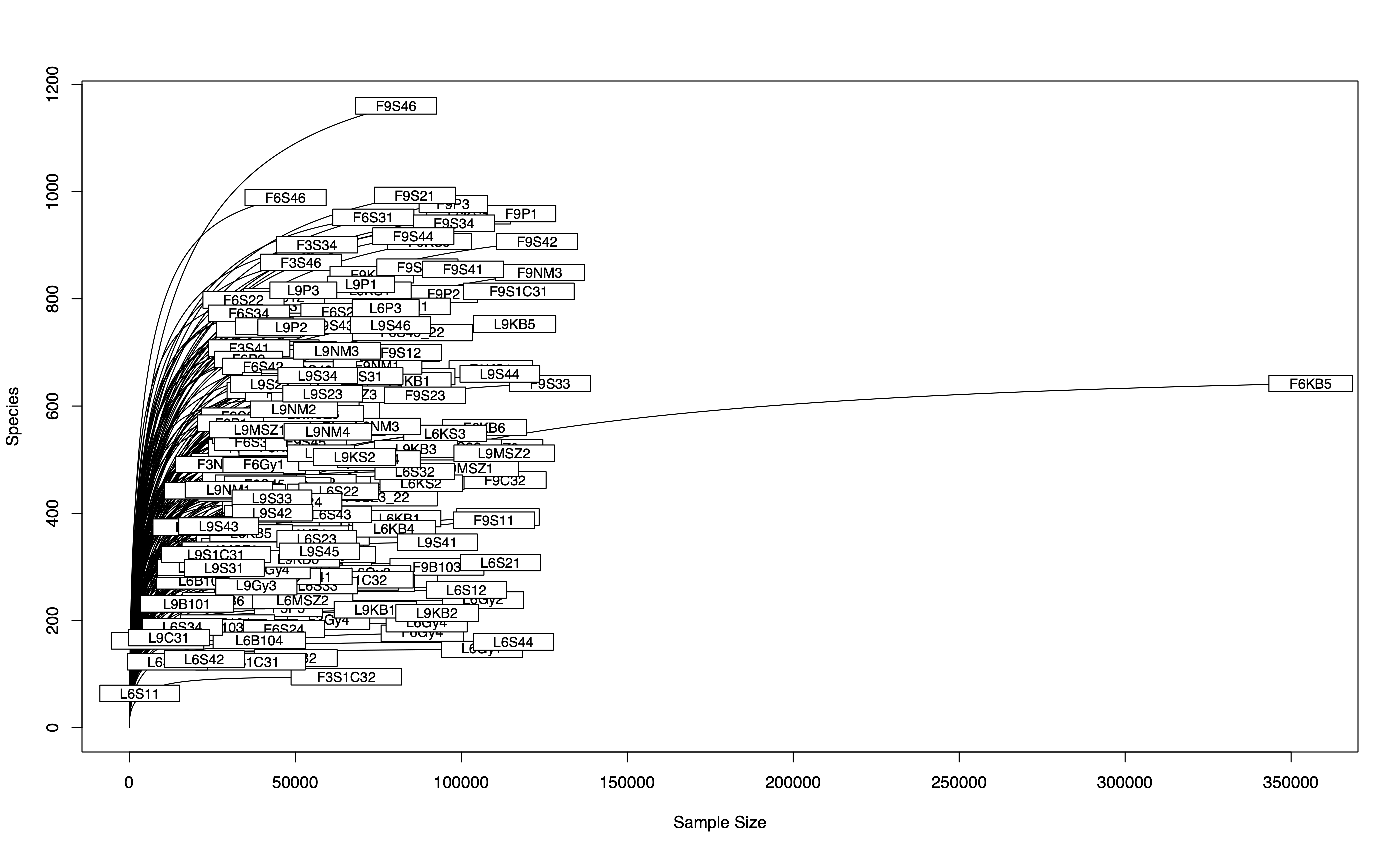

Supplement: Supplementary file 1 [file plants-14-03178-s001.zip › Supplementary_S5.tiff]
